# Development of Polyurethane/Peptide-Based Carriers with Self-Healing Properties

**DOI:** 10.3390/polym15071697

**Published:** 2023-03-29

**Authors:** Luiza Madalina Gradinaru, Maria Bercea, Alexandra Lupu, Vasile Robert Gradinaru

**Affiliations:** 1 “Petru Poni” Institute of Macromolecular Chemistry, 41-A Grigore Ghica Voda Alley, 700487 Iasi, Romania; 2 Faculty of Chemistry, “Alexandru Ioan Cuza” University, 11 Carol I Bd., 700506 Iasi, Romania

**Keywords:** polyurethane/peptide gel, self-assembly, self-healing carrier, amphiphilic polyurethane structure, viscoelastic behavior

## Abstract

In situ-forming gels with self-assembling and self-healing properties are materials of high interest for various biomedical applications, especially for drug delivery systems and tissue regeneration. The main goal of this research was the development of an innovative gel carrier based on dynamic inter- and intramolecular interactions between amphiphilic polyurethane and peptide structures. The polyurethane architecture was adapted to achieve the desired amphiphilicity for self-assembly into an aqueous solution and to facilitate an array of connections with peptides through physical interactions, such as hydrophobic interactions, dipole-dipole, electrostatic, π–π stacking, or hydrogen bonds. The mechanism of the gelation process and the macromolecular conformation in water were evaluated with DLS, ATR-FTIR, and rheological measurements at room and body temperatures. The DLS measurements revealed a bimodal distribution of small (~30–40 nm) and large (~300–400 nm) hydrodynamic diameters of micelles/aggregates at 25 °C for all samples. The increase in the peptide content led to a monomodal distribution of the peaks at 37 °C (~25 nm for the sample with the highest content of peptide). The sol–gel transition occurs very quickly for all samples (within 20–30 s), but the equilibrium state of the gel structure is reached after 1 h in absence of peptide and required more time as the content of peptide increases. Moreover, this system presented self-healing properties, as was revealed by rheological measurements. In the presence of peptide, the structure recovery after each cycle of deformation is a time-dependent process, the recovery is complete after about 300 s. Thus, the addition of the peptide enhanced the polymer chain entanglement through intermolecular interactions, leading to the preparation of a well-defined gel carrier. Undoubtedly, this type of polyurethane/peptide-based carrier, displaying a sol–gel transition at a biologically relevant temperature and enhanced viscoelastic properties, is of great interest in the development of medical devices for minimally invasive procedures or precision medicine.

## 1. Introduction

During the last decade, intensive efforts have been devoted to the development of soft materials, such as hydrogels, to design novel medical devices for minimally invasive procedures or precision medicine [1]. For instance, polymeric hydrogels with stimuli-responsive properties or self-healing abilities play an important role in the scaffold’s development due to their similar natural tissues. Stimuli-responsive polymers, also known as smart polymers, are one category of soft materials that respond to environmental changes such as temperature, pH, electric or magnetic fields, light, etc., and are widely used in biomedical applications [2,3]. Self-healing polymeric systems are smart 3D physical or chemical reversible networks that exhibit the ability to recover their structure after repeated damage, restoring the original functionality. Therefore, stimuli-responsive and self-healing hydrogels have achieved significant success in the development of various scaffolds used in the biomedical field such as drug carriers, wound dressings, tissue engineering, and so on [4,5]. The main driving forces that are employed in self-assembly molecules and self-healing polymers are the intermolecular interactions such as hydrogen bonding, hydrophobic forces, van der Waals forces, metal-ligand complexations, π–π interactions and electrostatic effects, together with their synergetic interactions [6,7]. The synergy of these forces could be explored in order to create supramolecular hydrogels with well-controlled properties that could play a fundamental role in the development of new therapies in various areas of interest, especially in the development of the next generation of targeted drug delivery carriers [5,8].

Generally, polyurethanes are a very versatile class of polymers with structures that can be engineered, due to the accurate selection of the building blocks, having good properties and wide applications in different areas of interest, such as automotive industries [9], painting [10], coatings [11,12], textiles [13], aeronautical [14] and petrochemical industries [15], marine applications [16,17], and so on. Due to their superior physical properties and high biocompatibility, these materials are also used in biomedical fields: in prosthesis development [18], tissue engineering [19], drug delivery systems [20], etc. The versatile chemistry of polyurethanes allows the incorporation of various hydrophilic/hydrophobic segments and specific functional groups into the polymeric backbone, which are able to customize their properties, in order to develop new multifunctional materials for a wide variety of applications [21,22,23]. The amphiphilic polyurethanes could self-assemble into micelles in an aqueous medium, a reversible and dynamic process useful for encapsulating hydrophobic, hydrophilic, or amphiphilic agents, providing them with versatile controlled delivery features. Moreover, these self-assembly structures can improve the stability of various bio-therapeutic agents including peptides, proteins, oligonucleotides, etc. to extend their circulation time in the body. An important issue in self-assembly systems is their capability to respond to an external stimulus, such as temperature, pH, electric or magnetic fields, ionic strength, or biochemical agents. Taking into consideration all of these aspects, we started to prepare some amphiphilic polyurethane structures based on poly(ethylene oxide)/poly(propylene oxide)/poly(ethylene oxide) (PEO–PPO–PEO) triblocks (e.g., Pluronic P123) as soft segments. This polyol was selected as a result of its particular quality, its aqueous solutions display a lower critical solution temperature (LCST), leading to a transition from a low-viscosity solution to a solid gel when they are heated [24]. Moreover, due to their commercial availability, non-cytotoxicity, high solubility, good biocompatibility, and excellent pharmacokinetics, these polymeric surfactants have been studied over time, in a broad range of applications, including drug delivery [25], gene transfection studies [26], tissue engineering [27], as coating agents [28], in theranostic devices [29], etc. Usually, Pluronics are unable to completely cover the surface of the drug particles, even when a structure with a large hydrophobic segment is used (e.g., P123 with hydrophilic-lipophilic balance < 20 and a PPO chain > 60). Another drawback of these Pluronic-based thermogels is their weak mechanical properties, such as low tensile strength and low Young modulus, which limit their practical applications. To overcome the drawbacks of Pluronics, different strategies have been proposed over time. Thus, some literature studies focused on the reaction of various Pluronics with diisocyanates, leading to the preparation of versatile polyurethane-based gels suitable for biomedical applications, especially in drug delivery formulations. For instance, valuable research work was performed by Boffito’s group, which reported the synthesis of thermosensitive gels based on Pluronic P407 extended with an aliphatic diisocyanate (i.e., 1,6-hexamethylene diisocyanate) and an amino acid-based diamine (i.e., L-Lysine ethyl ester-based diamine) [30], or an amino acid derived diol (i.e., N-Boc Serinol) [31]. In a recent work, Colucci et al. developed a multicomponent platform comprising a P407-based polyurethane embedded with curcumin nanoparticles with potential application in tissue engineering [32]. Moreover, Laurano et al. studied the effect of P407 molecular weight distribution on the physical and chemical properties of the prepared polyurethane hydrogels, concluding that the macrodiol purity had an important role in their performance [33]. Other studies reported the synthesis of thermoreversible polyurethane structures starting from Pluronic F127 and multiple extenders [34,35,36]. Regarding the use of Pluronic P123 as macrodiol for the synthesis of polyurethanes, few works are reported in the literature. Thus, Volkmer et al. extended Pluronic P123 with different diisocyanates (i.e., 1,4-butane diisocyanate; 1,6-hexamethylene diisocyanate or hydrogenated diphenylmethane diisocyanate) and the resulted gel was successfully implanted in vivo [37]. In previous publications by our group, different polyurethane-based hydrogels were developed and the influence of components used in synthesis on the final physical and chemical properties was investigated [22,38,39,40,41]. However, in the present study, we increased the hydrophobic segments by reaction with an aliphatic diisocyanate and extended with a bifunctional derivative of phosphatidylcholine leading to an amphiphilic polyurethane carrier system.

Generally, peptides are required for many biochemical processes and play an important role in fundamental physiological processes [42]. They are typically prepared by enzyme-assisted cleavage of proteins derived from natural sources, but they have several drawbacks, including poor reproducibility of gel properties due to the variations in the natural source, poorly defined molecular compositions, and an inability to control their chemistry to optimize the properties concerning the application [43]. Therefore, many researchers are exploring the potential of synthetic peptide preparation, especially with self-assembly properties to create gels that could be suitable for a broad range of biomedical applications [44,45]. However, most of these peptide-based gels are mechanically weak, limiting their use in biomedical applications, which require superior mechanical properties. To enhance the mechanical stability of the peptide-based gels, many strategies were developed, such as the incorporation of biomacromolecules or inorganic composites, physical, chemical, or enzymatic crosslinking, etc. [46]. To this end, we chose a collagen-inspired octapeptide sequence that exhibits self-assembly behavior and could mimic the structure and function of the natural extracellular matrix, allowing interaction with cells via biochemical signals [47]. Moreover, collagen is the most abundant protein in the body, widely used in biomedical applications due to its functional and structural diversity [48,49].

Specific interactions between peptides, proteins, or polymer molecules have been widely exploited over time for designing new smart responsive materials. Thus, by combining the self-assembly blocks of amphiphilic polyurethane structure with some inspired peptide, novel gels could be designed which could be explored as drug carriers for targeted delivery. Various polyurethane-peptide formulations have been developed, to control the degradation [50] or to induce the bioactivity [51,52] of the material.

Due to the increased number of various structures, it has become easy to design self-assembling molecules with controllable nanostructures for predictable biological functions and desired biomedical applications. In light of the aforementioned findings, the main goal of this study was to prepare novel polyurethane/peptide-based gels that could be designed as a platform to promote a sustained release of different drugs in the body. To the best of our knowledge, no works have been reported so far regarding the preparation of such a gel platform starting from an amphiphilic thermoreversible polyurethane structure and peptide. To achieve this goal, a versatile amphiphilic polyurethane-based system was synthesized using Pluronic P123 as a soft segment and 1,6-hexamethylene diisocyanate and (bis[2-(2-hydroxy-ethyldimethylammoniu)ethyl](polyethylenoxy)diphosphate) as a hard segment. Then, the peptide was added to the polyurethane solution and the impact on the self-assembly and self-healing properties was investigated. Based on the presented key findings, it is expected that this novel carrier could be an extremely useful approach that might be used as theragnostic agent, a powerful delivery carrier for hydrophobic drugs, or as matrices for repairing and regenerating different tissues.

## 2. Materials and Methods

### 2.1. Materials

Poly(ethylene oxide)–poly(propylene oxide)–poly(ethylene oxide) (PEO_20_–PPO_70_–PEO_20_) or Pluronic P 123 (Pl) with M_n_ = 5.8 × 10^3^ g/mol was purchased from Sigma Aldrich (Steinheim, Germany) and used as received. The bifunctional derivative of phosphatidylcholine, bis[2-(2-hydroxy-ethyldimethylammoniu)ethyl](polyethylenoxy)diphosphate (PC) with M_n_ = 5.92 × 10^2^ g/mol was a gift from Dr. O. Petreus (Department of Polycondensation and Thermostable Polymers, “P. Poni” Institute of Macromolecular Chemistry, Iasi, Romania). 1,6-Hexamethylene diisocyanate (HDI) was also obtained from Sigma Aldrich (Steinheim, Germany) and was freshly distilled before synthesis. The octapeptide (96.29% purity) with the sequence Asp-Val-Cys-Tyr-Tyr-Ala-Ser-Arg was purchased by Proteogenix, France. Dimethyl sulfoxide (DMSO) and N,N-dimethylformamide (DMF) were also supplied by Sigma Aldrich (Steinheim, Germany). Milli-Q water (18.2 MW∙cm) was produced by an Integrity+ Ultrapure water purification system (Adrona, Riga, Latvia). All other chemicals and reagents were of analytical grade and used as received without further purification.

### 2.2. Synthesis of Amphiphilic Polyurethane

The amphiphilic polyurethane (APU) was synthesized using the classical polyaddition reaction, according to our previously reported studies [39,53]. Briefly, one equivalent of Pluronic P 123 (Pl) was dehydrated at 100 °C and 0.1 mmHg for 2 h in a three-necked glass reactor equipped with a stirrer, a heating oil bath, a dropping funnel, and an N_2_ inlet and outlet. Then, the temperature was lowered to 80 °C and the reactor was brought to atmospheric pressure with purified N_2_. The required amount of HDI (2 equivalents) was added dropwise to the melt and stirred for 4 h to prepare the NCO-terminated polyurethane prepolymer. The prepolymer was then reacted with one equivalent of PC as a chain extender at 60 °C for 2 h, until the characteristic -NCO signal at around 2200 cm^−1^ in the infrared (IR) spectrum disappeared. This reaction was carried out at a molar ratio NCO:OH of 1:1.

### 2.3. Preparation of Polyurethane/Peptide-Based Carriers

An aqueous solution of 20% APU was prepared by mixing a predefined weight of polymer with Millipore water under vigorous mechanical stirring at room temperature, for 6 h, and then the solution was kept at 4 °C for 24 h until complete dissolution and homogenization. A stock solution of 8 mg/mL peptide in DMSO was also freshly prepared. We used DMSO to prepare the peptide solution because it is slightly soluble in an aqueous medium. Moreover, it was shown that a low concentration of DMSO did not evoke any substantial cytotoxic effect on the studied cells or it was used as a cryoprotectant for long-term preservation [54,55,56]. The polyurethane/peptide carriers were prepared by blending 20% APU solutions with different amounts of peptide solutions in a mass ratio of APU:peptide = 1000:1; 500:1 and 250:1 (denoted as P1, P2, P3, respectively). The final concentration of DMSO in the formulations was 10%. All the solutions were well homogenized, and all aqueous solutions used in various assays were prepared in Milli-Q water. The samples P1, P2, and P3 were investigated in similar conditions with the APU sample without peptide, denoted M.

### 2.4. Characterization Methods

#### 2.4.1. Attenuated Total Reflectance-Fourier Transform Infrared (ATR-FTIR) Spectroscopy

ATR-FTIR spectra were recorded using a Bruker Vertex 70 type spectrometer (Bruker, Germany), equipped with a diamond ATR device (Golden Gate, Bruker, Germany), and provided with software for spectral processing. Spectra were obtained in absorbance mode in the range of 4000–500 cm^−1^, averaging over 64 scans at a resolution of 2 cm^−1^. The spectra of the raw materials were recorded at room temperature. To study the effect of temperature on gelation, the solutions were heated at 37 °C, and equilibrated for 5 min prior to recording their spectrum using a Specac’s High-Temperature Golden Gate™ ATR Accessory. To distinguish characteristic bands of the sample gels, the spectra were obtained by subtracting the solvent component measured under the same conditions.

#### 2.4.2. Dynamic Light Scattering (DLS) Measurements

DLS measurements were performed using a Malvern Zetasizer (633 nm laser He/Ne) apparatus (Zetasizer model Nano ZS, Malvern Instruments, Malvern, UK). The system uses non-invasive back scatter (NIBS) technology to reduce the effects of multiple scattering. The measurements were performed in the range 0.6 nm–6 µm and the Mie method was applied. DLS measurements yield the Z-average of the aggregate’s apparent hydrodynamic diameter, calculated from diffusion coefficients using the Stokes-Einstein equation. The hydrodynamic diameter was expressed by intensity means, calculated from the signal amplitude. Zeta potential was determined using the Henry and Smoluchowski equation [57] based on electrophoretic mobility. The conductivity was determined during the acquisition of the zeta potential values. The samples were diluted in MilliQ water at a concentration of 0.1%. Their micellization behavior was studied at room (25 °C) and body temperatures (37 °C). An average of over three consecutive runs constitutes a measurement. Data analysis was performed with the Zetasizer software provided by Malvern, and the graphing was performed with OriginPro 8.5.

#### 2.4.3. Rheological Investigations

Rheological measurements were performed on a MCR 302 Anton-Paar rheometer (Gratz, Austria) equipped with plane-plane geometry of 25 mm (the selected gap was of 500 µm). The temperature was controlled by a Peltier device that allowed fast cooling and heating. To limit solvent evaporation, a device that creates a saturated atmosphere of solvent was used.

The gelation induced by temperature was investigated in an oscillatory shear regime for two heating rates, 0.5 °C/min and 1 °C/min, for temperatures ranging from 20 °C to 60 °C. The elastic (G′) and viscous (G″) moduli were determined, expressing the amount of stored and dissipated energy during one cycle of deformation, respectively. The loss tangent (tanδ) was determined as the ratio of viscous to elastic moduli and it is correlated with the viscoelasticity of the sample: tanδ < 1 for solid-like behavior and tanδ > 1 for liquid-like behavior.

The sample’s gelation at 37 °C was investigated using solutions stored at 25 °C and introduced then into the geometry of the rheometer. During the experiment, the temperature was first set at 25 °C for 120 s and then suddenly switched to 37 °C. The viscoelastic parameters G′, G″ and tanδ were monitored as a function of time at a constant oscillation frequency (ω) of 5 rad/s and strain amplitude (γ) of 1%.

The thermostated gels at 37 °C were investigated in different shear conditions. Amplitude sweep tests were carried out to determine the upper limit of strain, γ_L_, for the linear viscoelastic regime (Supporting Information Figure S1) and the yield stress value (σ_o_). A thixotropy test was carried out in strain steps oscillatory mode for ω = 5 rad/s and step strains successively varied every 300 s from low (1%) to high values (50%, 100%, 200%, 500%, and 1000% that belong to nonlinear range of viscoelasticity) and again the low step of strain (1%). The shear viscosity was determined at increasing and decreasing shear rates ($\dot{\gamma }$) from 0.01 s^−1^ to 1000 s^−1^. The creep and recovery behaviors were investigated by applying different constant shear stress values during the creep test (30 s) and then, by removing the action of shear stress, the strain recovery was monitored in time.

#### 2.4.4. Gel Permeation Chromatography (GPC)

The average molecular weight of APU was determined by GPC at 25 °C with a PL-EMD 950 evaporative mass detector instrument (Polymers Laboratories Ltd., Stretton, UK). Samples were eluted with DMF at a flow rate of 0.7 mL/min. Calibration was performed with narrow polydispersity polystyrene standards. Computer analysis of the elution data was based on the normalization of chromatograms.

### 2.5. Investigation of Gel Stability

The stability of gels was determined at 37 °C in PBS over 72 h. First, the gels were prepared by incubation at 37 °C for 10 min to allow the sol–gel transition and their initial weight (*W_i_*) was recorded. Then, 1 mL of PBS, previously equilibrated at 37 °C, was added to each sample and incubated for 1, 2, 3, 8, and 72 h. At each time interval, the remaining PBS was removed, the samples were weighted (*W_f_*), and fresh medium was added. At the end of the experiment, after 72 h of incubation, the samples were freeze-dried (ALPHA 1-2LD Martin Christ, Germany) and weighted (*W_f_-_dried_*) to estimate their mass loss. Control samples were also prepared by freeze-drying of gels that were not incubated in PBS (*W_i_-_dried_*). The tests were performed in triplicate. The absorption and stability of gels in the aqueous environment were quantified according to the following relations:

(1) $$PBS\;absorption\left(\%\right)=\left(W_{f}-W_{i}\right)/W_{f}\times 100$$

(2) $$mass\;loss\left(\%\right)=\left(W_{i\;dried}-W_{f\;dried}\right)/W_{i\;dried}\times 100$$

### 2.6. Statistical Analysis

The presented data are the average of triplicate experimental values and have the standard error of the mean. In addition, the statistical differences between data were performed using one-way analysis of variance (ANOVA). The *p* values < 0.05 was considered to be statistically significant.

## 3. Results and Discussion

### 3.1. Synthesis and Characterization of Amphiphilic Polyurethane

**Figure 1:**
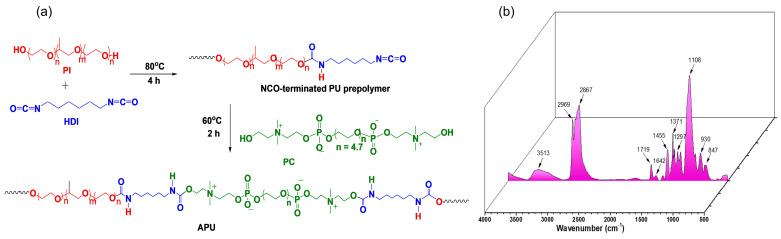
(**a**) Synthesis route and (**b**) IR spectrum of APU.

The typical synthesis route of the amphiphilic polyurethane (APU) is illustrated in Figure 1a. First, the polyol (Pl) was dried at temperature, under vacuum, to remove the moisture prior to the polyaddition reaction and then reacted with an excess of HDI to yield the isocyanate end-capped prepolymer. In the second step, PC was added as a chain extender to build a linear, ordered structure with alternating blocks of soft and hard segments. The reaction was carried out in bulk without a catalyst, at a molar ratio between -NCO and -OH of 1:1. As mentioned, we chose Pl as the soft segment of APU since its structure self-assembles into micelles in aqueous medium and when heated, its solution forms a solid gel. We selected HDI as the hard segment precursor because it is a linear aliphatic diisocyanate that is widely used in the synthesis of biocompatible polyurethane structures [58]. The chain extension step also has a significant impact on the final polyurethane properties, not just due to the structure and concentration of the extender, but also due to process variables that influence the particles size distribution [59]. Thus, to increase the density of the intra- and intermolecular interactions between hard domains, a bifunctional derivative of phosphatidylcholine (PC) was chosen. Its chemical structure has a negatively charged phosphate linkage and a positively charged ammonium moiety (Figure 1a) that could enhance the intermolecular interactions. Furthermore, this is a derivative of phosphatidylcholine, a naturally occurring lipid that is a fundamental building block of plasma cell membranes, with many potential applications in biomedicine [60].

These two types of domains—soft and hard—tend to phase-separate, leading to the formation of some microdomains. The resulting polyurethane chains consist of alternating sequences of soft that are flexible chains and hard segments that act as physical crosslinks. The properties of the final polymer depend strongly on the degree of phase separation between hard and soft segments and the interconnectivity of the hard segments [58].

The preparation of the APU was monitored by infrared spectroscopy, as shown in Figure 1b. The spectrum showed the characteristic bands ascribed to the urethane bonds, as described below. The -OH and -NH absorption bands were observed in the range 3678–3152 cm^−1^, with a maximum peak at 3513 cm^−1^. The asymmetric and symmetric stretching vibrations of aliphatic groups appeared at 2969 and 2867 cm^−1^, respectively. The absorption bands at around 1719 and 1642 cm^−1^ belonged to the stretching vibrations of Amide I (C=O and C-NH groups, respectively) [22,61]. The peak at 1532 cm^−1^ is due to the overlapping of N–H flexural vibration and C–N stretching vibration (Amide II) [39,62].

The absorption bands at 1455, 1371, and 1310 cm^−1^ were assigned to -CH_2_ bending vibration, -C-H bending symmetric vibration and -CH_2_ wagging, respectively. Amide III was also identified at around 1236 cm^−1^ [63]. The P=O bond of phospholipid stretching was observed around 1249 cm^−1^ [64,65] and -P-O-CH_2_ bond at 1030 cm^−1^ [66]. C-O-C stretching vibrations were noticed in the range 1100–1000 cm^−1^. IR and ^1^H-NMR spectra (Supporting Information Figure S2) confirmed the successful preparation of APU. GPC was employed for the determination of the average molecular weights of APU, M_n_ = 19.66 kg/mol, and a polydispersity index of 1.2.

### 3.2. Development of Gels Based on APU and Peptide

The polyurethane/peptide-based gels were prepared by mixing a solution of 20% APU with peptide solutions in different molar ratios, as mentioned in the previous section. These macromolecules self-assemble in aqueous solutions due to the favorable physical interactions between the amphiphilic structure of polyurethane and peptide. The presence of peptide in the mixture would strengthen the inter- and intramolecular interactions such as hydrophobic interactions, dipole-dipole, electrostatic, π–π stacking or hydrogen bonds, leading to enhanced polymer chain entanglement. This formulation is temperature responsive, leading to gels with potential biomedical applications, such as carriers for controlled drug delivery in the body.

**Figure 2:**
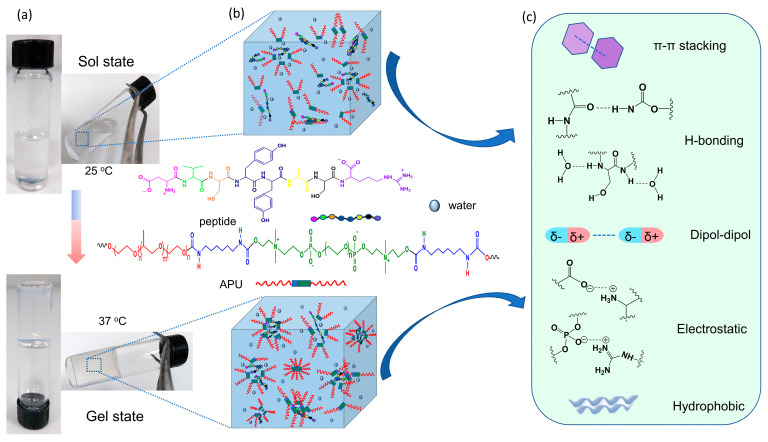
Illustration of self-assembly behavior of polyurethane/peptide-based gel: (**a**) Optical images of tub inverted test containing samples at 25 °C and 37 °C; (**b**) Self-assembling representation in sol and gel states; (**c**) Some physical inter- and intramolecular interactions during gel formation.

The visual observation of the phase transition behavior of the polyurethane/peptide aqueous solutions was first demonstrated by the tube inverted test, as shown in Figure 2a. Initially, the samples appeared as transparent solutions at room temperature (~25 °C) and changed into gels when the temperature increased to 37 °C. Therefore, at low temperatures, the polyurethane and peptide move freely in water and can easily interact with each other. Then, with the increasing temperature, the self-assembling process takes place due to favorable physical interactions between water and polymeric macromolecules, leading to a more packed, 3D crosslinked network in the gel state (Figure 2b,c). Since no chemical change occurs, the gelation is completely reversible, and when the gel is cooled down, it returns to the solution state.

Thus, this dual behavior as solution at room temperature and gel at body temperature makes these polymer carriers attractive for the development of medical devices for minimally invasive procedures or precision medicine. Moreover, these in situ formed polyurethane/peptide-based carriers could be easily injected into the body using a 21G gauge needle.

### 3.3. Study of the Gelation Process

To investigate the mechanism of the gelation process, different approaches, such as DLS, ATR-FTIR, and rheology were chosen to study the polyurethane-peptide conformation evolution in an aqueous environment at room (~25 °C) and body (37 °C) temperatures.

#### 3.3.1. DLS Investigations

**Figure 3:**
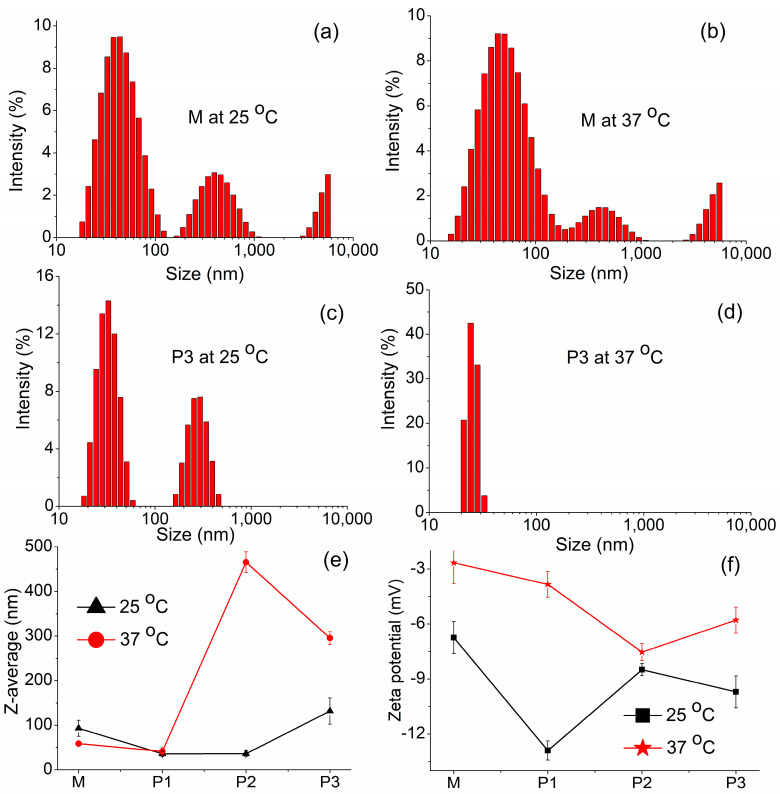
Dynamic light scattering data of polyurethane/peptide-based carriers: (**a**–**d**) Size distribution of micelles for M and P3 samples at 25 and 37 °C, respectively; The influence of the polyurethane/peptide mass ratio on the (**e**) Z-average and (**f**) zeta potential at 25 and 37 °C.

As a first step, we evaluated the phenomenology of the micelle formation process using DLS measurements, which seems to be a highly effective technique for investigating the dynamics of macromolecular assembly in solution. Moreover, size, size distribution, and stability of micelles in solution are important factors to achieve optimum clinical outcomes or to evaluate the stability of the developed formulations in storage [67]. The size distribution of micelles and aggregates is illustrated in Figure 3a–d for M (peptide-free formulation) and P3 (formulation with the highest amount of peptide) samples in solution, at 25 and 37 °C, respectively. The parameters for all the samples monitored by the DLS technique, such as the hydrodynamic diameter of the micelles/aggregates (DH), mean diameter, polydispersity index (PDI), zeta potential or conductivity, are summarized in Table 1. The micellar distribution revealed an anisometric system, especially at room temperature. This is a result of stronger hydrophobic interactions between the segments from APU structure compared to hydrophilic segments [39,40]. The bimodal distribution peaks indicated that two types of micelles/aggregates were formed in solution. If we analyzed the peptide-free sample (M) at room temperature, it was found that small micelles with a hydrodynamic diameter of around 47 nm coexisted with much larger micelles or aggregates of 440 nm in diameter. When the temperature was raised to 37 °C, the aggregation and packing interactions between micelles increased and the hydrodynamic diameter slightly increased. In the sample with the highest amount of peptide (P3), the same bimodal distribution of the micelles or aggregates was observed at room temperature, but with a relatively narrow size distribution. At 37 °C, both types of micelles/aggregates were bridged together to form micellar groups resulting in a monomodal distribution of the peaks. The monodisperse profile may suggest that a single, packed type of micelles/aggregates was formed in their aqueous mixtures. This phenomenon was also observed for sample P2 (Table 1).

The change in the micelle’s diameter with the addition of peptide is better highlighted in Figure 3e, where the Z-average as a function of peptide amount is illustrated. At 25 °C, a shift of the Z-average toward smaller sizes around 35–36 nm can be observed for the first two samples (P1 and P2) when compared with M, but P3 presents a higher size of micelles or aggregates, around 130 nm. This is perhaps due to the decrease in the packing possibility of the macromolecular chains when the peptide amount increases, leading to high micelles/aggregates. The polydispersity index (PDI) represents the dimensional homogeneity and distribution of the micelles in the solution [67]. Thus, values exceeding 0.7 indicate a broad size distribution, whereas lower values suggest a mono-dispersed sample with better homogeneity [40,68]. Our investigated samples presented small values between 0.2 and 0.35 at room temperature, which can be regarded as a uniform size distribution. When the temperature was increased to 37 °C slightly greater values up to 0.79 were noticed. This suggests that micelles/polymicelles and aggregates coexist in the solution. Furthermore, it was shown that small molecules with phenolic hydroxyl groups exhibit a high impact on the micellar morphology of Pluronic P123. The locations of small molecules within the micelles are influenced by the balance of hydrogen bonds and hydrophobic interactions between small molecules and the copolymer [69].

The stability of micelles in solution through electrostatic repulsion between them can influence the zeta potential. While this parameter quantifies the electric field potential of the micelles, it is affected by the size, shape and surface charge of the structure [70]. However, micelles aggregation requires a low absolute value of zeta potential. The variation of the zeta potential with the peptide amount in formulations is illustrated in Figure 3f and Table 1. Zeta potential presented a decrease in the values with the increase in the peptide amount, indicating a low surface charge due to the interactions between the amphiphilic polyurethane chains and peptide functional groups. It was also observed that the rise in the temperature induces an increase in the zeta potential values, meaning that the micelles and aggregates are restructured at 37 °C. The conductivity values are well correlated with the zeta potential values. In conclusion, the addition of the peptide in the polyurethane solutions led to strong interactions between macromolecular chains, resulting in good folding and packing of macromolecules in gels.

#### 3.3.2. ATR-FTIR Spectroscopy Analysis

The heat-induced gelation was further studied by ATR-FTIR spectroscopy, a suitable and sensitive technique for observing changes due to the interactions and bonding states of functional groups.

**Figure 4:**
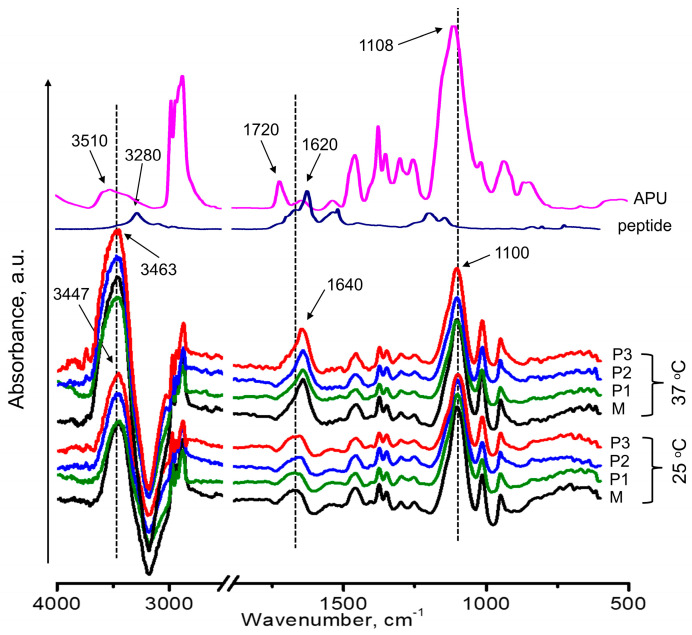
ATR-FTIR spectra of solutions (25 °C) and gels (37 °C) for the investigated samples compared with raw APU and peptide spectra.

Figure 4 presents the ATR-FTIR spectra of the polyurethane/peptide samples in sol (25 °C) and gel (37 °C) states. The spectra of the APU and peptide were also shown for comparison. In the sol state, at 25 °C, it was observed that the spectra present typical bands characteristic to the APU and peptide structures, but with significant variations regarding the intensity and shift of the peaks. Therefore, if we compare the bands that correspond to the -OH stretching vibration of APU with that of its solutions, it can be seen that the intensity increases and shifts to lower wavelengths. Furthermore, all the peaks displayed a blue shift due to the inter- and intramolecular H-bonding interactions between water molecules from solvent and the functional groups of the macromolecular chains of polyurethane and peptide. Furthermore, the bands corresponding to the C=O stretching vibrations are larger than in the raw APU structure.

Regarding the influence of the peptide concentration in solutions, no relevant differences can be highlighted in IR spectra, as is illustrated in Figure 4. This is probably due, on one hand, to the small mass ratio peptide/polyurethane (1:1000–1:250) or, on the other hand, to the high amount of the solvent (water) in the gel (80%).

When the solutions were heated to 37 °C, some changes in the IR spectra were observed due to the formation of the gels. First, the intensity of the bands is enhanced in the gel state, especially at the bands between 33,600 and 3300 cm^−1^ and 1760 and 1650 cm^−1^ corresponding to the stretching vibrations of -OH and C=O. Second, the IR bands are more clearly defined and narrowed, suggesting that the interactions between the involved macromolecules are stronger in the gel state. If in the sol state, the water and polymeric macromolecules move freely, during the sol–gel transition, the hydrogen bonding changes, due to the dehydration of hydrophobic domains, and some polymer-polymer interactions take place. Now, the movement of polymer segments is restricted by the gelation process, resulting in a three-dimensional, physically crosslinked structure. These results confirmed the formation of a more organized structure, with a more packed network in the gel state [39].

**Figure 5:**
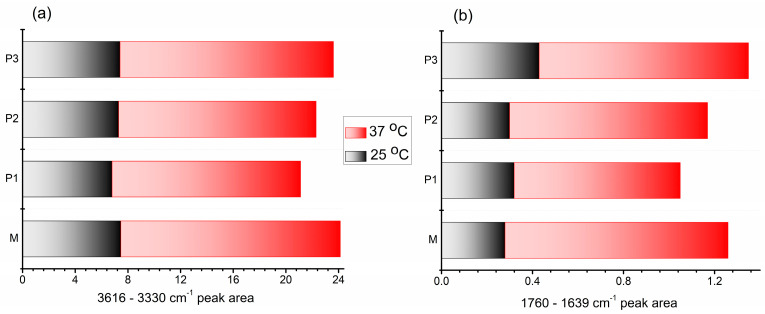
Variation of the peak area between (**a**) 3616 and 3330 cm^−1^ and (**b**) 1760 and 1639 cm^−1^ as a function of increasing temperature for the investigated samples.

The -OH/-NH and -C=O stretching vibrations can be markedly influenced by physical interactions such as hydrogen bonding, where the band position is determined by the backbone conformation and the hydrogen-bonded pattern. Both bands are sensitive to the strength of hydrogen bonding and the analysis of these regions allows emphasizing these changes [71]. Therefore, the peak area evolution as a function of temperature and composition was calculated and the results are illustrated in Figure 5. Both specific vibration bands showed an increasing value of the area in the gel state, at 37 °C, compared to that of the corresponding solution. These results further demonstrated that the temperature increase contributes to enhanced physical crosslinking to form compact gel networks. If in the variation of the -OH peak’s area (Figure 5a), no significant changes were observed regarding the influence of the peptide amount, a small increase was revealed with the increase of the peptide content, in the variation of the carbonyl peak’s area (Figure 5b). This indicated that the introduction of peptide segments increases the physical crosslinking between the involved molecules, leading to a more compact structure with enhanced properties.

#### 3.3.3. Rheological Behavior

**Figure 6:**
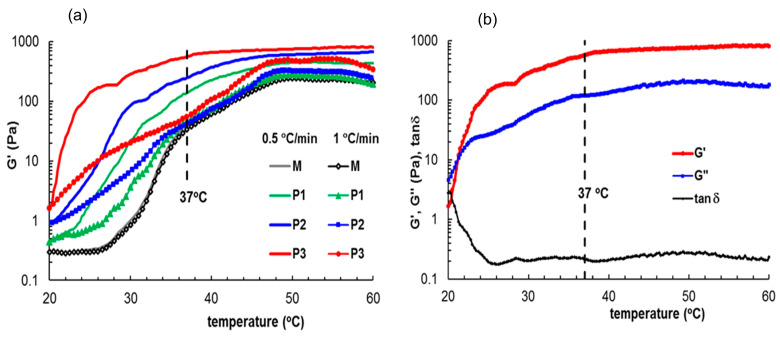
G′ variation during gelation induced by temperature increase at different heating rates (**a**) 1 °C/min (full symbols) and 0.5 °C/min (lines); (**b**) viscoelastic parameters for sample P3 as a function of temperature for a heating rate of 0.5 °C/min.

Keeping in mind the perspective of further development of this carrier for use as medical gels, the dynamic rheological behavior by oscillatory rheometry was evaluated, with a particular emphasis on the properties at 37 °C. The attractive interactions between the components of these structures may strongly impact the rheological properties. Thus, the gelation of this polyurethane/peptide-based carrier induced by the temperature increase was followed for two heating rates, 0.5 °C/min and 1 °C/min, as illustrated in Figure 6. According to Figure 6a, the heating rate has a very small influence on sample M (peptide-free formulation). However, the kinetics of gelation and sol–gel transition points are sensibly influenced according to the peptide concentration.

At 20 °C, all samples presented a micellar structure (well above the critical micelle concentration (CMC); for example, the CMC of M sample was 0.6 × 10^−6^ mol/L as determined by surface tension measurements [39]) and liquid-like behavior with G′ < G″ and tanδ > 1. The micelles/aggregates contain a hydrophobic core and hydrophilic shell, which present an extended conformation due to favorable interactions with the solvent. Then, the physical interactions increase as the temperature is raised. The samples undergo a sol–gel transition which is accompanied by a sharp variation in the viscoelastic parameters beyond the transition point (considered to be the temperature for which G′ = G″). In the gel state, G′ was greater than the G′’ indicating that the elastic properties predominate over the viscous ones. The transition temperature and gelation kinetics are influenced by the sample composition and heating rate (Table 2). The presence of peptide determines the occurrence of other types of intra- and intermolecular interactions such as dipole-dipole, electrostatic, π–π stacking or hydrogen bonds, leading to enhanced polymer chain entanglement (Figure 2). Therefore, the viscoelastic moduli and complex viscosity increase with the peptide content during gelation. In the gel state, the differences between the samples are diminished, but the network strength increases with peptide content (Table 2).

**Figure 7:**
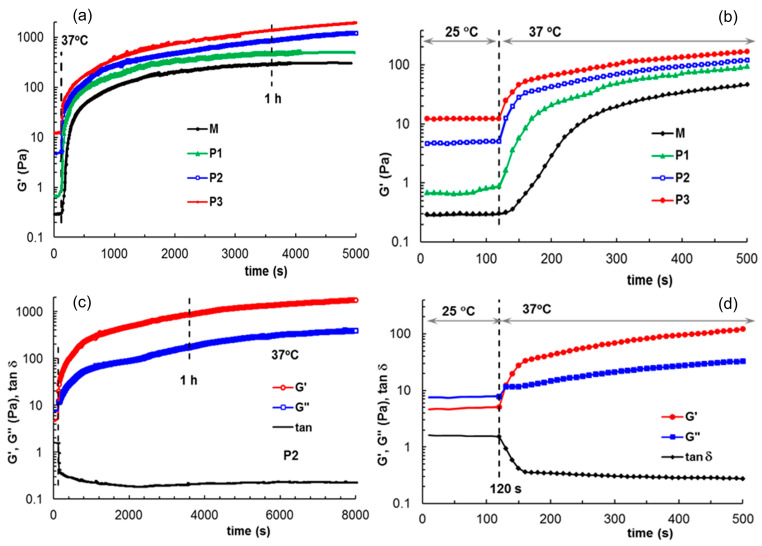
(**a**,**b**) Evolution of G′ during gelation at 37 °C for all investigated samples; (**c**,**d**) Viscoelastic parameters G′, G″ and tanδ during gelation for sample P2. The samples were first tested at 25 °C for 120 s, then the temperature was suddenly switched to 37 °C, and in situ gelation was monitored through the rheological parameters. (**b**,**d**) detail the behavior of the systems at the beginning of gelation.

In situ gelation was monitored for thermostated samples at 25 °C (room temperature), introduced into the rheometer and submitted to oscillatory deformation at this temperature for 120 s for a clear evidence of the sol state (Figure 7). The temperature was suddenly changed to 37 °C and the gelation kinetics was monitored through the viscoelastic parameters. The sol–gel transition occurs very fast for all samples (within 20–30 s) but the equilibrium state of the thermal induced gel structure is attained after 1 h for samples M and P1. For samples P2 and P3, more than 1 h is necessary to reach the equilibrium. The network structure progressively formed by polymers and peptides during gelation at 37 °C is influenced to a high extent by the sample composition, as shown by the values of the viscoelastic moduli (G′ and G″), the upper limit of strain for the linear viscoelastic range (γ_L_) or yield stress (σ_o_)(Table 2). The yield stress, σ_o_, is defined as the highest values of shear stress for which the rheological response is still elastic [72] and for σ > σ_o_ the flow starts. All these parameters (G′, G″, γ_L_, σ_o_) increase with the peptide content, suggesting that the gel samples are more structured in the presence of peptide.

The use of thermosensitive gels is an advantageous route for bioprinting or injectable gels because they present low viscosity in sol state and can be easily printed or administered with a syringe to the targeted place/tissue where the in situ formed structure ensures the desired shape.

### 3.4. Self-Healing Behavior of Gels Illustrated in Different Rheological Tests

Self-healing carriers, i.e., carriers that restore structural properties after deformation and failure, are gaining attention from the research community since there is a constant need to extend the lifespan, improving the safety and performance of materials, especially in biomedical applications [73]. The self-healing behavior is due to the dynamic re-build of the relatively weak bonds such as hydrogen bonds, ionic interactions, etc. that were damaged by an external stimulus [74]. Moreover, the self-healing ability is a requirement for bioinks or injectable biomaterials [75,76,77]. After flowing through a syringe needle, the network structure is strongly disturbed, and the structural integrity must be recovered in order to maintain the functionality of biomaterials. Moreover, the shear forces applied to gels during their use affect the cell viability [6,78,79]. In such cases, soft materials with moderate yield stress and softness, shear thinning, and self-healing behavior represent appropriate choices.

**Figure 8:**
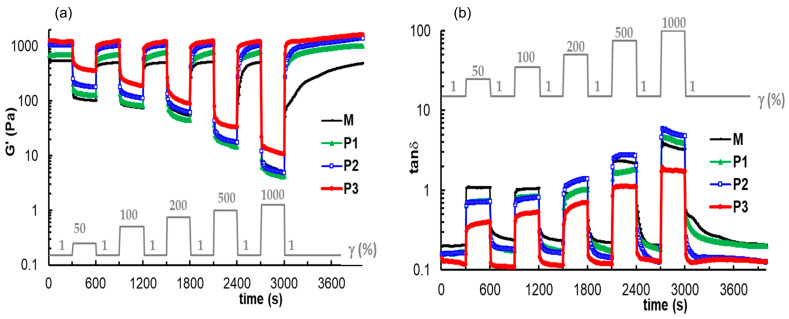
The elastic modulus (**a**) and loss tangent (**b**) during the experiments at low and high successive step strains applied every 300 s.

The self-healing ability of the prepared gels was analyzed by performing some rheological tests that offer information concerning the recovery of the structures. First, the thixotropy of gel samples was investigated in oscillatory shear experiments for ω = 5 rad/s, the step strains were switched successively each 300 s from low amplitude (γ = 1%) to a high amplitude value and again to low γ value of 1%. The high level of strains was selected each time in the nonlinear viscoelastic regime: 50%, 100%, 200%, 500%, and 1000%. During these experiments, the parameters G′, G″ and tanδ were monitored as a function of time (Figure 8). Sample M is characterized by a very good recovery after the first three cycles of deformation. The required time for recovering the initial structure increases as the strain increases, from 30 s after the first cycle with γ = 50% to 200 s after the fourth cycle (γ = 500%). Finally, for the highest level of deformation, which was tested experimentally, the structure is recovered after more than 2000 s. The timescale for re-building the original structure of the gel (the equilibrium state before shearing) is different for hybrid gels. In the presence of peptide, the structure recovery after each cycle of deformation is a time-dependent process, and the recovery is complete after about 300 s. However, the structural integrity is affected to a lesser extent by the high strains applied to the peptide-containing gels compared to the M sample. The structure recovery after a large deformation is not instantaneous, but the interactions involved in the network structure are re-established relatively quickly—in about 300 s. This small delay in structure recovery represents an important advantage in bioprinting when sudden gel restructuring can affect the cell viability [75]. According to Figure 8 and the data presented in Table 2, the optimum behavior for the investigated samples in the present study is shown in the P2 formulation: the gelation starts at room temperature, the network strength is close to the P3 gel, and the structure recovery occurs in about 300 s after the sample was subjected to large deformations.

**Figure 9:**
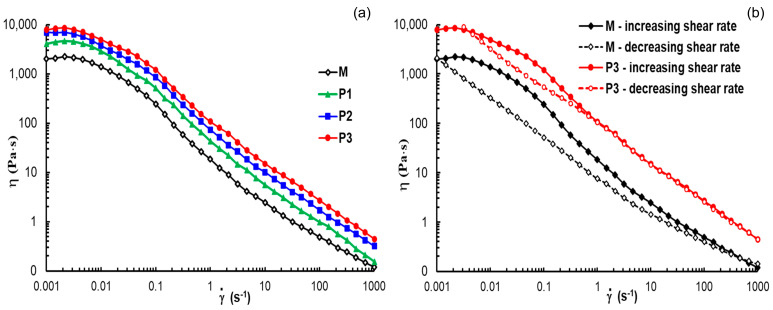
Plots of apparent viscosity as a function of shear rate for all investigated samples in stationary shear conditions (**a**) at increasing shear rate; (**b**) curves of apparent viscosity for samples M and P3 at increasing (full symbol) and decreasing (open symbol) shear rate.

Another rheological test that provides information about the recovery of the structures is the shear flow behavior. Figure 9 shows the variations in apparent viscosity with the applied shear rate for each investigated gel sample. At low shear rates, the Newtonian viscosity was registered and its value increases with peptide concentration (Table 2). The transition to a non-Newtonian region occurs over more than a decade and this is due to different structural entities present in the sample (micelles, polymicelles, aggregates) that start progressively to flow. Above 0.1 s^−1^, in the non-Newtonian region, the viscosity scales as η ~ ${\dot{\gamma }}^{-n}$, where the flow index *n* has values around 0.81. This suggests that the gels behave as entangled networks due to the interactions developed in hybrid systems above the gelation temperature.

For all samples, shear thinning behavior is attained during flow from very low shear rates (below 0.01 s^−1^, Figure 9a). By applying high shear forces, the gel structure is disturbed, and the structural units are in flow state; the gel structure is progressively recovered at decreasing shear rate, describing a hysteresis loop (Figure 9b). This represents another possibility to investigate the thixotropic behavior, by following the structure breakdown under high shear rate conditions and then the structure build-up by diminishing the shear forces. The area of the hysteresis loop significantly decreases by the addition of the peptide into formulation (Table 2), due to a faster structure recovery in the presence of the peptide.

**Figure 10:**
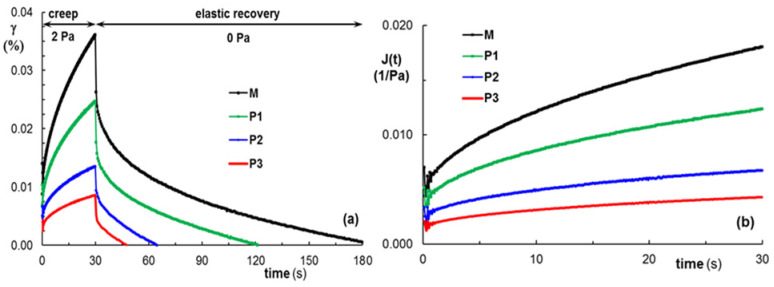
The behavior of gel samples (M, P1, P2 and P3) during creep at 2 Pa (**a**) strain during creep and recovery tests (**b**) J(t) during creep tests.

The viscoelasticity of gels can be discussed through the creep and recovery curves (Figure 10). This behavior, associated with a high loss modulus in relation to elastic modulus, represents an important characteristic, especially for the gels used for cell entrapment [74]. The samples were submitted to constant shear stress (σ) for a period of time *t* = 30 s (creep curve, Figure 10a) when a time-dependent strain γ(t) is registered. Then, the shear stress is removed, and the recovered strain is followed until the equilibrium state is attained (recovery curve, Figure 10a). During the creep test, γ(t) is correlated with the creep compliance, J(t) (Figure 10b):

γ (t) = σJ(t)(3)

**Figure 11:**
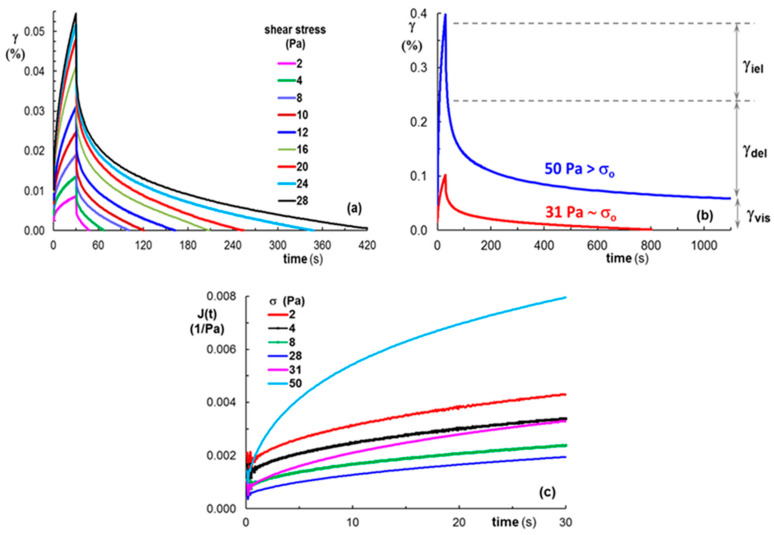
(**a**,**b**) Strain and (**c**) creep compliance during creep and recovery tests for P3 gel sample submitted successively to increasing shear stress values.

The gel samples were submitted to different shear stress values below and above the yield stress (Figure 11). During the creep test, the applied stress determines a transient response, which includes the instantaneous elastic strain (γ_iel_), the delayed elastic strain (γ_del_), and the permanently viscous part (γ_vis_). When the action of shear stress is stopped, γ_iel_ is first recovered, followed by γ_del_, whereas γ_vis_ registered at equilibrium is irreversible (Figure 11b). For low shear stress values (as for example 2 Pa, Figure 10a) all gels are able to recover the entire amount of deformation, γ_vis_ = 0. However, the recovery time depends on the sample’s ability to restore the initial structure and it decreases as the peptide content increases (Figure 10a). By increasing the applied shear stress to values below σ_o_, the total deformation increases (at *t* = 30 s), and the recovery time also increases (Figure 11a). The permanent (viscous) deformation of the gels was observed for σ > σ_o_ (Figure 11b), where the structure recovery takes a very long time.

By applying shear stress below σ_o_, the creep compliance J(t) decreases with increasing shear stress; around σ_o_, J(t) no longer respects this monotonous decrease, and it begins to increase with the shear stress (Figure 11c), suggesting the presence of viscous flow.

When the shear stress is below σ_o,_ the curves from Figure 10a and Figure 11a clearly illustrate the complete recovery of strain, as for viscoelastic solids. The multiple physical interactions established between the polyurethane and peptide, as hydrogen bonds between urethane and amide, as well as phenolic or serine moieties, along with ionic interaction between phosphate moieties and positively charged residue from peptide N- and C-terminus, make these structures behave as versatile viscoelastic gels at 37 °C, with an ability to self-heal and shear thinning behavior, making them suitable for injectable materials or 3D printing.

### 3.5. Investigation of Gel Stability at 37 °C in an Aqueous Environment

**Figure 12:**
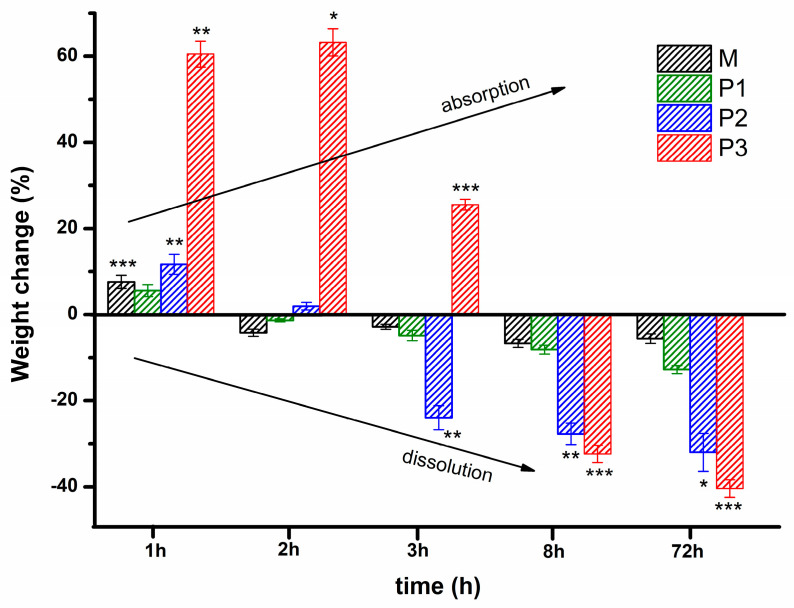
Stability profile for polyurethane/peptide-based carriers at 37 °C in PBS. Statistical significance: *** *p* < 0.001; ** 0.001 < *p* < 0.01; * 0.01 < *p* < 0.05.

Considering the possibility of further development of the polyurethane/peptide-based carrier as an injectable gel for biomedical applications, the stability in PBS at 37 °C, as a function of time, was evaluated. This method evaluates the change in weight after the incubation of the gel samples in PBS medium at body temperature, being a suggestive factor for the assessment of swelling as well as dissolution/erosion [30,31,33,35]. Thus, the stability profile over time is illustrated in Figure 12. This shows that all the gels are able to dissolve in an aqueous environment after a certain period of time. After one hour of incubation, the samples were stable and presented a maximum swelling degree of around 7, 5, 11, and 60% for M, P1, P2, and P3, respectively. Then, they progressively lost their weight with an increase in the incubation time. The sample with the highest amount of loading peptide (P3) was stable and still swelled up to 65% even after 2 h of incubation. After 3 h, the swelling degree of P3 decreases up to 25% and then a negative change in weight was observed, suggesting that gel dissolution occurred. At 8 h of incubation until the end of the experiment (72 h), the gels showed a progressive mass loss with increasing peptide amount. All the samples presented a further negative change in weight data, indicating that the dissolution had completely overcome the swelling. After 72 h of incubation, when the experiment was stopped, the samples were freeze-dried, and the percentage of mass loss was calculated. The remaining mass after drying was 24, 22, 11, and 6% for M, P1, P2, and P3, respectively. Thus, the mass loss progressively increases with the loading amount of peptide, after 8 h of incubation in PBS. Therefore, the addition of such a peptide structure led to an increase in the swelling ability in the first 2 h of incubation in PBS at 37 °C for the sample with the highest amount of peptide, followed by a large increase in mass loss up to 72 h. This could be explained by the interplay of physical interactions of various functional groups from gel structures, especially when new polar groups (-OH, -NH-CO-) of peptide are present. These additional groups led to the enhancement of hydrogen bond networks with water molecules from an aqueous environment.

## 4. Conclusions

In situ-forming gels are promising materials for biomedical applications, since they provide the possibility of being used as injectable carriers due to their sol–gel transition at a biologically relevant temperature. To this end, a polyurethane/peptide-based carrier was developed from an amphiphilic polyurethane and a triple charged peptide. This peptide sequence was selected because it is found in human collagen type IV, a major component of membranes. Moreover, it is specifically from the noncollagenous domain that confers specificity to the chain assembling. Furthermore, it could mimic the structure and function of the natural extracellular matrix, allowing further various weak interactions with cells. Thus, the unique molecular structure of the amphiphilic polyurethane and the incorporation of peptide were responsible for the self-assembling in the aqueous medium, due to a synergy of many favorable physical interactions. The presence of peptide in the formulation determines strengthening of the inter- and intramolecular interactions such as hydrophobic interactions, dipole-dipole, electrostatic, π–π stacking, or hydrogen bonds, leading to enhanced chain entanglements. The mechanism of the gelation process was evaluated through DLS, ATR-FTIR, and rheological measurements at room and body temperatures. These investigations revealed that the addition of the peptide in the polyurethane solution leads to strong interactions between different macromolecular chains, resulting in good folding and packing in the network.

The self-healing ability of the prepared gels was analyzed by performing some rheological tests that offered information concerning the recovery of the structures after applying high external forces. The recovery of the rheological parameters (G′, G′’) to their original values after removing the repetitive high strain, demonstrates the reversibility and stability of the self-healing response of the polyurethane/peptide gels. This self-healing could be attributed to the physical network that enables the macromolecules to quickly reorganize.

In conclusion, the prepared polyurethane/peptide carrier is a versatile material with low viscosity in sol state at room temperature, and viscoelastic properties typical to gels at body temperature. Furthermore, this material exhibits self-healing ability and shear thinning behavior, the required properties for injectable materials or 3D printing.

These results demonstrate that the investigated formulation presents a versatile structure and has great potential as a carrier for the targeted delivery of hydrophobic drugs or other molecules. This study opens a wide range of possibilities for specific applications in the area of drug delivery or tissue regeneration, where self-assembly and self-healing mechanisms are extremely valuable attributes. Therefore, this research provides a new pathway in the development of tailored medical devices for minimally invasive procedures or precision medicine.

## Figures and Tables

**Table 1:** DLS parameters characteristic to the polyurethane/peptide carriers.

Sample Code	Temperature (°C)	D_H_ (nm)	D_H_ (nm)	Z-Average (nm)	PDI	Zeta Potential (mV)	Conductivity (mS/cm)
		Peak 1	Peak 2				
M	25	46.91 ± 19.53	439.2 ± 168.1	93.04 ± 17.91	0.206	−6.75 ± 0.87	0.0112
	37	56.42 ± 30.11	440.6 ± 173.7	58.72 ± 4.93	0.799	−2.66 ± 1.14	0.0125
P1	25	30.76 ± 10.84	295.2 ± 81.94	35.87 ± 5.76	0.318	−12.9 ± 0.52	0.0184
	37	33.99 ± 9.52	221.3 ± 46.63	41.25 ± 8.06	0.255	−3.84 ± 0.7	0.00943
P2	25	35.32 ± 14.8	321.6 ± 76.26	36.03 ± 6.8	0.359	−8.49 ± 0.33	0.00842
	37	21.23 ± 2.34	-	465.5 ± 23.27	0.406	−7.53 ± 0.46	0.0203
P3	25	32.86 ± 8.03	282.9 ± 66.74	131.7 ± 29.51	0.256	−9.7 ± 0.87	0.00502
	37	25.26 ± 2.99	-	295.3 ± 14.76	0.641	−5.79 ± 0.71	0.00568

**Table 2:** Gelation temperature and some rheological characteristics of gel samples at 37 °C.

Sample Code	T_gelation_ ^a^ (°C)	T_gelation_ ^a^ (°C)	G′ ^b^ (Pa)	G″ ^b^ (Pa)	γ_L_ ^c^ (%)	σ_o_ ^c^ (Pa)	η_o_ (Pa·s)	*n*	Thixotropic Area (Pa·s)
	(0.5 °C/min)	(1 °C/min)							
M	29.4	34	547	110	2.16	20.6	2220	−0.829	128.67
P1	27.5	31	696	119	4.59	21.2	4690	−0.815	72.56
P2	25.8	27.6	1030	139	9.89	28.3	6980	−0.817	51.16
P3	21.2	23.5	1180	153	10.23	55.5	8600	−0.816	49.22

^a^ temperature sweep test, γ = 1%; ω = 5 rad/s; ^b^ γ = 1%, ω = 5 rad/s; ^c^ amplitude sweep test, ω = 5 rad/s.

## Data Availability

The data presented in this study are available on request from the corresponding author.

## Supplementary Materials

The following supporting information can be downloaded at: https://www.mdpi.com/article/10.3390/polym15071697/s1, Figure S1: Variation of viscoelastic moduli for gel samples in amplitude sweep test at 37 °C (ω = 5 rad/s); Figure S2: ^1^H-NMR spectrum of APU.
