# Supplementary material for: Development of Polyurethane/Peptide-Based Carriers with Self-Healing Properties
Source: Polymers (Basel). 2023 Mar 29;15(7):1697. doi: 10.3390/polym15071697 (PMC10096672; doi:10.3390/polym15071697)
Supplement: Supplementary file 1 [file polymers-15-01697-s001.zip › polymers-2279346-supplementary.pdf]

polymers

MDPI

Supplementary Materials

Development of Polyurethane/Peptide-Based Carriers with Self-Healing Properties

Luiza Madalina Gradinaru [1,*], Maria Bercea [1], Alexandra Lupu [1] and Vasile Robert Gradinaru [2]

1 "Petru Poni" Institute of Macromolecular Chemistry, 41-A Grigore Ghica Voda Alley, 700487 Iasi, Romania; bercea@icmpp.ro (M.B.); lupu.alexandra@icmpp.ro (A.L.)
2 Faculty of Chemistry, "Alexandru Ioan Cuza" University, 11 Carol I Bd., 700506 Iasi, Romania; robert.gradinaru@uaic.ro
* Correspondence: gradinaru.luiza@icmpp.ro

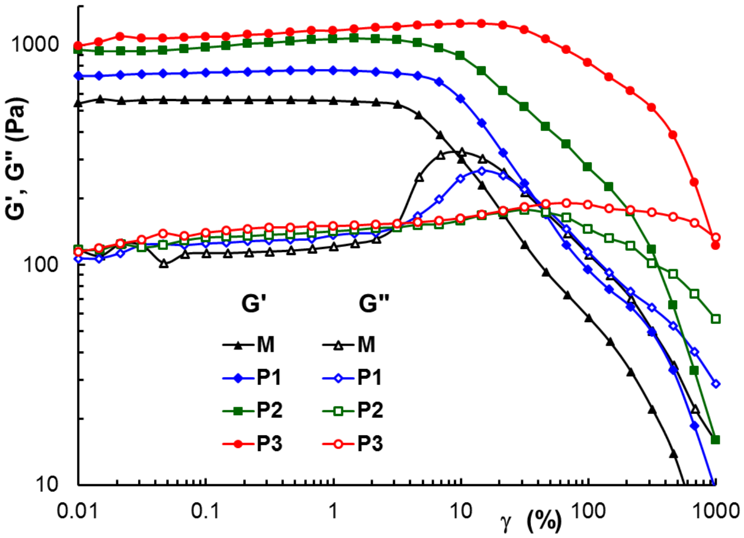

Figure S1. Variation of viscoelastic moduli for gel samples in amplitude sweep test at 37 °C (ω = 5 rad/s).

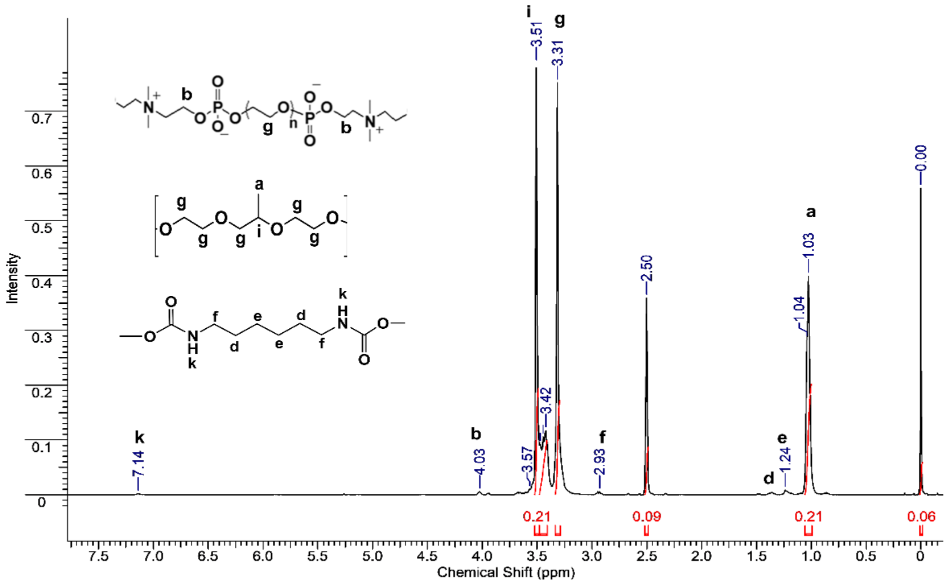

Figure S2. ^{1}H-NMR spectrum of APU.
